# Sodium *p*-toluenesulfinate tetra­hydrate

**DOI:** 10.1107/S1600536811021738

**Published:** 2011-06-11

**Authors:** Richard Betz, Thomas Gerber

**Affiliations:** a Nelson Mandela Metropolitan University, Summerstrand Campus, Department of Chemistry, University Way, Summerstrand, PO Box 77000, Port Elizabeth, 6031, South Africa

## Abstract

The title compound, Na^+^·C_7_H_7_O_2_S^−^·4H_2_O, is the hydrate of the sodium salt of *para*-toluene­sulfinic acid. The mol­ecular geometry around the sulfur atom is tetra­hedral with X–S–Y angles spanning a range of 102.23 (6)–110.04 (6)°. In the crystal, the water mol­ecules connect the sodium cations into chains along the *b* axis via O—H⋯O hydrogen bonds. An inter­molecular O—H⋯π inter­action is also observed.

## Related literature

For the crystal structure of sodium *para*-toluene­sulfonate, see: Reinke & Rudershausen (1999 ▶). For details of graph-set analysis of hydrogen bonds, see: Etter *et al.* (1990 ▶); Bernstein *et al.* (1995 ▶).
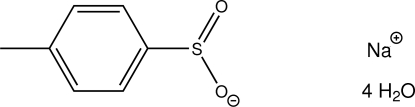


## Experimental

### 

#### Crystal data

Na^+^·C_7_H_7_O_2_S^−^·4H_2_O
*M* *_r_* = 250.24
Monoclinic, 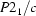

*a* = 15.9432 (19) Å
*b* = 6.1825 (7) Å
*c* = 12.2668 (15) Å
β = 100.166 (5)°
*V* = 1190.1 (2) Å^3^
*Z* = 4
Mo *K*α radiation
μ = 0.31 mm^−1^
*T* = 200 K
0.53 × 0.39 × 0.21 mm

#### Data collection

Bruker APEXII CCD diffractometer
Absorption correction: multi-scan (*SADABS*; Bruker, 2008 ▶) *T*_min_ = 0.826, *T*_max_ = 1.000
10741 measured reflections
2846 independent reflections
2554 reflections with *I* > 2σ(*I*)
*R*_int_ = 0.025

#### Refinement

*R*[*F* ^2^ > 2σ(*F* ^2^)] = 0.029
*wR*(*F* ^2^) = 0.087
*S* = 1.11
2846 reflections
162 parameters
12 restraints
H atoms treated by a mixture of independent and constrained refinement
Δρ_max_ = 0.72 e Å^−3^
Δρ_min_ = −0.23 e Å^−3^

### 

Data collection: *APEX2* (Bruker, 2010 ▶); cell refinement: *SAINT* (Bruker, 2010 ▶); data reduction: *SAINT*; program(s) used to solve structure: *SHELXS97* (Sheldrick, 2008 ▶); program(s) used to refine structure: *SHELXL97* (Sheldrick, 2008 ▶); molecular graphics: *ORTEPIII* (Farrugia, 1997 ▶) and *Mercury* (Macrae *et al.*, 2008 ▶); software used to prepare material for publication: *SHELXL97* and *PLATON* (Spek, 2009 ▶).

## Supplementary Material

Crystal structure: contains datablock(s) I, global. DOI: 10.1107/S1600536811021738/dn2695sup1.cif

Supplementary material file. DOI: 10.1107/S1600536811021738/dn2695Isup2.cdx

Structure factors: contains datablock(s) I. DOI: 10.1107/S1600536811021738/dn2695Isup3.hkl

Supplementary material file. DOI: 10.1107/S1600536811021738/dn2695Isup4.cdx

Additional supplementary materials:  crystallographic information; 3D view; checkCIF report

## Figures and Tables

**Table 1:** Hydrogen-bond geometry (Å, °) *Cg* is the centroid of the C1–C6 ring.

*D*—H⋯*A*	*D*—H	H⋯*A*	*D*⋯*A*	*D*—H⋯*A*
O3—H31⋯O1	0.81 (1)	1.98 (1)	2.7885 (14)	173 (2)
O3—H32⋯O2^i^	0.81 (1)	2.02 (1)	2.8059 (16)	166 (2)
O4—H41⋯O2	0.80 (1)	2.16 (1)	2.9038 (15)	154 (2)
O4—H42⋯O3^ii^	0.81 (1)	1.99 (1)	2.7884 (15)	173 (2)
O5—H51⋯O1^iii^	0.80 (1)	1.99 (1)	2.7760 (15)	167 (2)
O5—H52⋯O2^iv^	0.80 (1)	2.16 (1)	2.9319 (15)	163 (2)
O6—H61⋯O1^v^	0.80 (1)	2.21 (2)	2.9528 (17)	154 (2)
O6—H62⋯*Cg*^vi^	0.79 (1)	2.89 (2)	3.3782 (17)	122 (2)

Symmetry codes: (i) 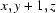; (ii) 
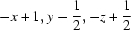
; (iii) 
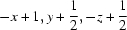
; (iv) 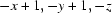; (v) 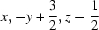; (vi) 
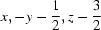
.

**Fig. 1.**
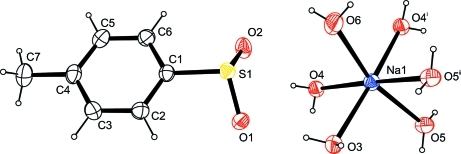
The molecular structure of the title compound, with atom labels and anisotropic displacement ellipsoids (drawn at 50% probability level). Symmetry operators: i -x + 1, -y + 1, -z; ii -x + 1, -y + 2, -z. For reasons of clarity, only one of the sodium cations with its octahedral coordination of water molecules is depicted instead of the polymeric chain.

**Fig. 2.**
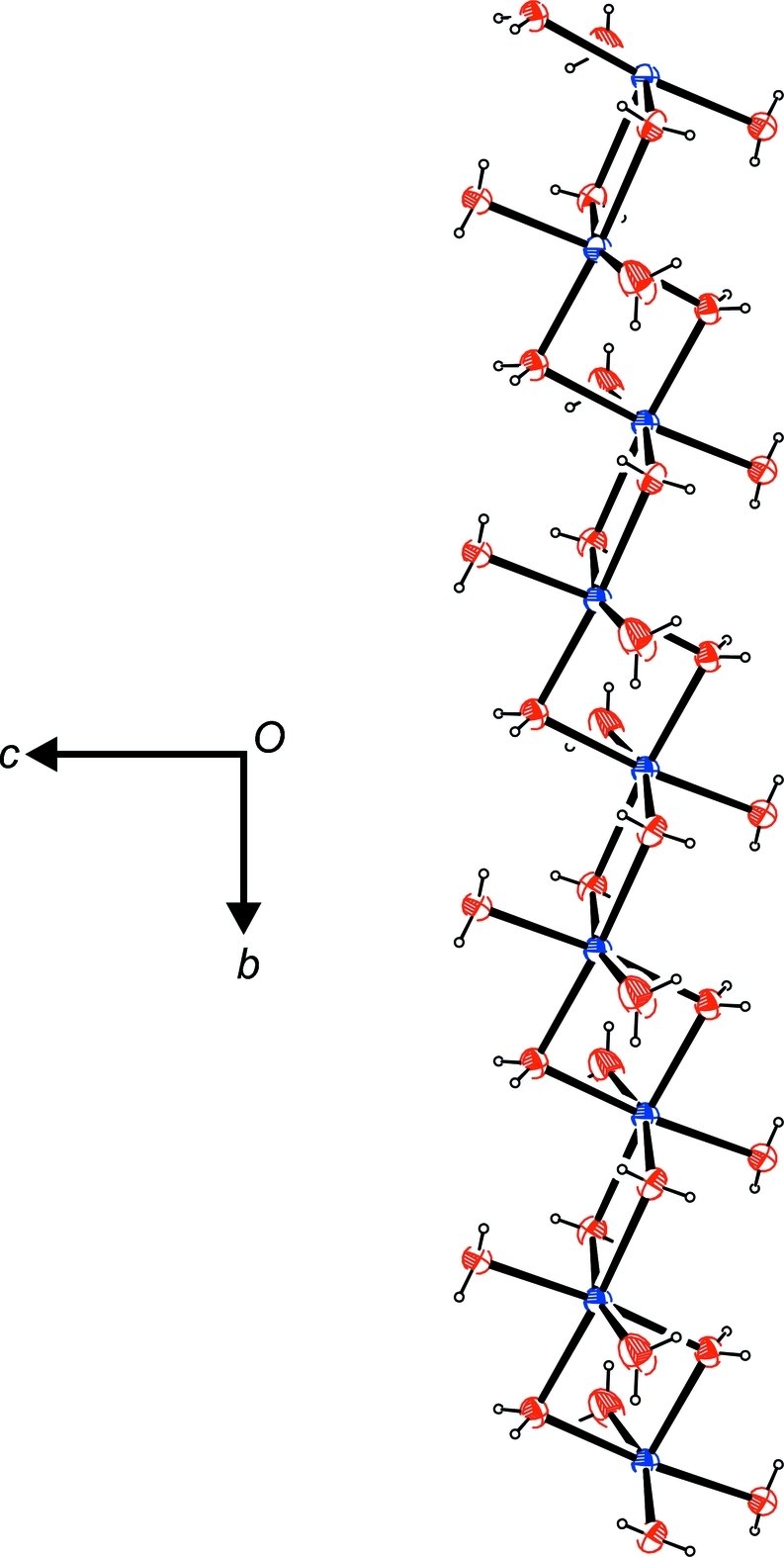
Polymeric chain of sodium cations and water molecules, viewed along [1 0 0].

## supplementary crystallographic information

### Comment

Multidentate ligands play a major role in the synthesis of coordination polymers and metal-organic framework compounds (MOFs). Especially derivatives of benzoic acid have found widespread use in this aspect and a variety of these coordination polymers have been characterized in solution and in the solid state. Owing to the desire to synthesize functionalized MOFs whose poresizes or even complete architectural set-ups might easily be influenced upon variation of external parameters such as pH value or the presence and concentration of molecules that might reside inside the pores of these MOFs, chelating ligands related to benzoic acid but with the ability to change their bonding behaviour are necessary. In this aspect, *para*-toluenesulfinic acid seemed of interest since it may act as neutral or anionic ligand, and even the sulfur atom may show donor action. In order to gather structural information to allow for the tailored synthesis of MOFs based on *para*-toluenesulfinic acid, we determined the crystal structure of its sodium salt. The crystal structure of the sodium salt of *para*-toluenesulfonic acid is apparent in the literature (Reinke & Rudershausen, 1999).

Taking into account the lone pair on the sulfur atom, the latter is present in a pseudo-tetrahedral molecular geometry. The *X*–S–Y angles span a range of 102.23 (6)–110.04 (6) °. The least-squares planes defined by the atoms of the aromatic system on the one hand and the SOO group on the other hand intersect at an angle of 64.47 (6) °. The sodium cation is coordinated by six water molecules (two of them symmetry-generated) of which two act as bridging ligands to the neighbouring sodium cation and thus foster the formation of a "sodium-acqua-polymer" chain along the crystallographic *b* axis (Fig. 2). The angles between two *trans*-orientated water molecules in the resultant [Na(H_2_O)_6_]^+^ octahedra were found adopting values between 163.96 (5) ° and 173.68 (4) °.

The crystal structure is dominated by hydrogen bonds. Except for one of the hydrogen atoms on one water molecule that is part of a O–H···π interaction, all of the water molecules take part in O–H···O hydrogen bonding. Each of the sulfinic acid group's O atoms acts as multifold acceptor. In terms of graph set analysis (Etter *et al.* (1990); Bernstein *et al.* (1995)), the description of the hydrogen bonding systems necessitates a *DDDDDDDD* descriptor on the unitary level. In total, the components of the crystal structure are connected to double layers perpendicular to the crystallographic *a* axis with the hydrophobic aromatic moieties forming the outer surfaces of these layers. π-Stacking is not a prominent feature of the crystal structure with the shortest distance between two aromatic systems found at 5.5359 (11) Å.

### Experimental

The compound was obtained commercially (KEG). Crystals suitable for the X-ray diffraction study were obtained upon free evaporation of an aqueous solution thereof.

### Refinement

Carbon-bound H-atoms were placed in calculated positions (C—H 0.95 Å) and were included in the refinement in the riding model approximation, with *U*(H) set to 1.2*U*_eq_(C). The H-atoms of the water molecules were located on a difference Fourier map, and their O—H distances as well as their H–O–H angles were refined using *DFIX* instructions with one common free variable, with their *U*(H) set to 1.5*U*_eq_(O).

### Crystal data

**Table:**

Na^+^·C_7_H_7_O_2_S^−^·4H_2_O	*F*(000) = 528
*M**_r_* = 250.24	*D*_x_ = 1.397 Mg m^−^^3^
Monoclinic, *P*2_1_/*c*	Mo *K*α radiation, λ = 0.71073 Å
Hall symbol: -P 2ybc	Cell parameters from 7792 reflections
*a* = 15.9432 (19) Å	θ = 2.6–27.4°
*b* = 6.1825 (7) Å	µ = 0.31 mm^−^^1^
*c* = 12.2668 (15) Å	*T* = 200 K
β = 100.166 (5)°	Platelet, colourless
*V* = 1190.1 (2) Å^3^	0.53 × 0.39 × 0.21 mm
*Z* = 4	

### Data collection

**Table:**

Bruker APEXII CCD diffractometer	2846 independent reflections
Radiation source: fine-focus sealed tube	2554 reflections with *I* > 2σ(*I*)
graphite	*R*_int_ = 0.025
φ and ω scans	θ_max_ = 28.0°, θ_min_ = 3.4°
Absorption correction: multi-scan (*SADABS*; Bruker, 2008)	*h* = −21→20
*T*_min_ = 0.826, *T*_max_ = 1.000	*k* = −5→8
10741 measured reflections	*l* = −16→16

### Refinement

**Table:**

Refinement on *F*^2^	Primary atom site location: structure-invariant direct methods
Least-squares matrix: full	Secondary atom site location: difference Fourier map
*R*[*F*^2^ > 2σ(*F*^2^)] = 0.029	Hydrogen site location: inferred from neighbouring sites
*wR*(*F*^2^) = 0.087	H atoms treated by a mixture of independent and constrained refinement
*S* = 1.11	*w* = 1/[σ^2^(*F*_o_^2^) + (0.0378*P*)^2^ + 0.535*P*] where *P* = (*F*_o_^2^ + 2*F*_c_^2^)/3
2846 reflections	(Δ/σ)_max_ = 0.001
162 parameters	Δρ_max_ = 0.72 e Å^−^^3^
12 restraints	Δρ_min_ = −0.23 e Å^−^^3^

### Fractional atomic coordinates and isotropic or equivalent isotropic displacement parameters (Å^2^)

**Table:**

	*x*	*y*	*z*	*U*_iso_*/*U*_eq_	
Na1	0.45418 (3)	0.75184 (9)	0.02242 (4)	0.02500 (14)	
O3	0.39909 (7)	0.88972 (17)	0.17974 (9)	0.0287 (2)	
H31	0.3681 (11)	0.796 (2)	0.1963 (17)	0.043*	
H32	0.3672 (11)	0.990 (2)	0.1611 (16)	0.043*	
O4	0.48457 (7)	0.41959 (17)	0.12242 (8)	0.0274 (2)	
H41	0.4404 (8)	0.378 (3)	0.1373 (15)	0.041*	
H42	0.5152 (10)	0.417 (3)	0.1824 (11)	0.041*	
O5	0.59271 (7)	0.89815 (18)	0.06769 (9)	0.0293 (2)	
H51	0.6176 (11)	0.927 (3)	0.1281 (11)	0.044*	
H52	0.6263 (11)	0.846 (3)	0.0338 (14)	0.044*	
O6	0.31527 (9)	0.6560 (3)	−0.07052 (11)	0.0501 (4)	
H61	0.2992 (17)	0.711 (4)	−0.1295 (14)	0.075*	
H62	0.2971 (16)	0.537 (2)	−0.072 (2)	0.075*	
S1	0.25275 (2)	0.41422 (5)	0.14718 (3)	0.02338 (10)	
O1	0.30441 (6)	0.54447 (17)	0.24003 (8)	0.0293 (2)	
O2	0.31037 (7)	0.26401 (18)	0.09734 (9)	0.0342 (2)	
C1	0.19547 (8)	0.2298 (2)	0.22163 (11)	0.0230 (3)	
C2	0.17035 (9)	0.2961 (2)	0.31990 (12)	0.0280 (3)	
H2	0.1867	0.4342	0.3504	0.034*	
C3	0.12128 (9)	0.1588 (3)	0.37294 (12)	0.0306 (3)	
H3	0.1042	0.2048	0.4396	0.037*	
C4	0.09673 (8)	−0.0448 (2)	0.33007 (12)	0.0287 (3)	
C5	0.12217 (9)	−0.1073 (2)	0.23143 (13)	0.0298 (3)	
H5	0.1056	−0.2451	0.2006	0.036*	
C6	0.17114 (9)	0.0278 (2)	0.17743 (12)	0.0271 (3)	
H6	0.1879	−0.0178	0.1105	0.032*	
C7	0.04458 (11)	−0.1935 (3)	0.38969 (15)	0.0422 (4)	
H7A	0.0714	−0.2048	0.4678	0.063*	
H7B	0.0416	−0.3372	0.3555	0.063*	
H7C	−0.0131	−0.1349	0.3845	0.063*	

### Atomic displacement parameters (Å^2^)

**Table:**

	*U*^11^	*U*^22^	*U*^33^	*U*^12^	*U*^13^	*U*^23^
Na1	0.0267 (3)	0.0233 (3)	0.0256 (3)	0.0000 (2)	0.0062 (2)	0.0000 (2)
O3	0.0328 (5)	0.0258 (5)	0.0283 (5)	−0.0027 (4)	0.0078 (4)	0.0004 (4)
O4	0.0267 (5)	0.0300 (5)	0.0259 (5)	−0.0014 (4)	0.0057 (4)	0.0009 (4)
O5	0.0277 (5)	0.0322 (6)	0.0275 (5)	−0.0005 (4)	0.0036 (4)	−0.0044 (4)
O6	0.0465 (7)	0.0606 (9)	0.0391 (6)	−0.0253 (7)	−0.0043 (5)	0.0124 (6)
S1	0.02337 (17)	0.02331 (18)	0.02327 (17)	−0.00030 (12)	0.00355 (12)	0.00159 (12)
O1	0.0332 (5)	0.0270 (5)	0.0266 (5)	−0.0075 (4)	0.0021 (4)	0.0002 (4)
O2	0.0374 (6)	0.0302 (5)	0.0403 (6)	−0.0011 (4)	0.0212 (5)	−0.0026 (4)
C1	0.0190 (6)	0.0247 (6)	0.0251 (6)	0.0006 (5)	0.0034 (5)	0.0016 (5)
C2	0.0284 (7)	0.0272 (7)	0.0291 (7)	−0.0020 (5)	0.0071 (5)	−0.0040 (5)
C3	0.0288 (7)	0.0365 (8)	0.0281 (7)	−0.0016 (6)	0.0093 (5)	−0.0014 (6)
C4	0.0210 (6)	0.0325 (7)	0.0325 (7)	−0.0016 (5)	0.0046 (5)	0.0046 (6)
C5	0.0261 (7)	0.0253 (7)	0.0377 (8)	−0.0026 (5)	0.0043 (6)	−0.0018 (6)
C6	0.0254 (6)	0.0276 (7)	0.0284 (6)	0.0000 (5)	0.0054 (5)	−0.0031 (5)
C7	0.0392 (9)	0.0421 (9)	0.0481 (9)	−0.0111 (7)	0.0153 (7)	0.0055 (8)

### Geometric parameters (Å, °)

**Table:**

Na1—O5	2.3604 (12)	S1—O2	1.5094 (11)
Na1—O6	2.3801 (13)	S1—O1	1.5142 (10)
Na1—O4	2.3977 (12)	S1—C1	1.8062 (14)
Na1—O3	2.4127 (12)	C1—C6	1.3893 (19)
Na1—O4^i^	2.4178 (12)	C1—C2	1.3971 (19)
Na1—O5^ii^	2.4843 (12)	C2—C3	1.391 (2)
Na1—Na1^ii^	3.4837 (11)	C2—H2	0.9500
O3—H31	0.810 (11)	C3—C4	1.394 (2)
O3—H32	0.807 (11)	C3—H3	0.9500
O4—Na1^i^	2.4178 (12)	C4—C5	1.397 (2)
O4—H41	0.801 (11)	C4—C7	1.512 (2)
O4—H42	0.809 (12)	C5—C6	1.389 (2)
O5—Na1^ii^	2.4843 (12)	C5—H5	0.9500
O5—H51	0.798 (11)	C6—H6	0.9500
O5—H52	0.802 (11)	C7—H7A	0.9800
O6—H61	0.799 (12)	C7—H7B	0.9800
O6—H62	0.792 (12)	C7—H7C	0.9800
			
?···?	?		
			
O5—Na1—O6	163.96 (5)	Na1—O5—Na1^ii^	91.92 (4)
O5—Na1—O4	96.42 (4)	Na1—O5—H51	126.7 (14)
O6—Na1—O4	96.81 (5)	Na1^ii^—O5—H51	106.2 (15)
O5—Na1—O3	97.70 (4)	Na1—O5—H52	114.0 (14)
O6—Na1—O3	91.82 (5)	Na1^ii^—O5—H52	107.3 (15)
O4—Na1—O3	87.86 (4)	H51—O5—H52	107.7 (16)
O5—Na1—O4^i^	81.86 (4)	Na1—O6—H61	116.7 (19)
O6—Na1—O4^i^	90.07 (5)	Na1—O6—H62	123.4 (19)
O4—Na1—O4^i^	85.93 (4)	H61—O6—H62	108 (2)
O3—Na1—O4^i^	173.68 (4)	O2—S1—O1	110.04 (6)
O5—Na1—O5^ii^	88.08 (4)	O2—S1—C1	102.39 (6)
O6—Na1—O5^ii^	79.76 (5)	O1—S1—C1	102.23 (6)
O4—Na1—O5^ii^	172.58 (4)	C6—C1—C2	119.84 (13)
O3—Na1—O5^ii^	85.68 (4)	C6—C1—S1	120.09 (10)
O4^i^—Na1—O5^ii^	100.59 (4)	C2—C1—S1	119.91 (10)
O5—Na1—Na1^ii^	45.46 (3)	C3—C2—C1	119.68 (13)
O6—Na1—Na1^ii^	121.63 (4)	C3—C2—H2	120.2
O4—Na1—Na1^ii^	141.52 (4)	C1—C2—H2	120.2
O3—Na1—Na1^ii^	92.13 (3)	C2—C3—C4	121.22 (13)
O4^i^—Na1—Na1^ii^	92.01 (3)	C2—C3—H3	119.4
O5^ii^—Na1—Na1^ii^	42.62 (3)	C4—C3—H3	119.4
O5—Na1—Na1^i^	88.79 (3)	C3—C4—C5	118.14 (13)
O6—Na1—Na1^i^	94.68 (5)	C3—C4—C7	120.58 (14)
O4—Na1—Na1^i^	43.19 (3)	C5—C4—C7	121.27 (14)
O3—Na1—Na1^i^	131.04 (4)	C6—C5—C4	121.35 (14)
O4^i^—Na1—Na1^i^	42.74 (3)	C6—C5—H5	119.3
O5^ii^—Na1—Na1^i^	143.21 (4)	C4—C5—H5	119.3
Na1^ii^—Na1—Na1^i^	123.83 (3)	C5—C6—C1	119.76 (13)
Na1—O3—H31	105.8 (15)	C5—C6—H6	120.1
Na1—O3—H32	110.1 (14)	C1—C6—H6	120.1
H31—O3—H32	103.7 (16)	C4—C7—H7A	109.5
Na1—O4—Na1^i^	94.07 (4)	C4—C7—H7B	109.5
Na1—O4—H41	106.7 (14)	H7A—C7—H7B	109.5
Na1^i^—O4—H41	120.8 (14)	C4—C7—H7C	109.5
Na1—O4—H42	121.2 (14)	H7A—C7—H7C	109.5
Na1^i^—O4—H42	113.7 (14)	H7B—C7—H7C	109.5
H41—O4—H42	101.5 (16)		
			
O5—Na1—O4—Na1^i^	−81.33 (4)	O1—S1—C1—C6	−151.45 (11)
O6—Na1—O4—Na1^i^	89.58 (5)	O2—S1—C1—C2	147.07 (12)
O3—Na1—O4—Na1^i^	−178.84 (4)	O1—S1—C1—C2	33.07 (12)
O4^i^—Na1—O4—Na1^i^	0.0	C6—C1—C2—C3	0.2 (2)
O5^ii^—Na1—O4—Na1^i^	151.6 (3)	S1—C1—C2—C3	175.65 (11)
Na1^ii^—Na1—O4—Na1^i^	−88.11 (6)	C1—C2—C3—C4	0.2 (2)
O6—Na1—O5—Na1^ii^	40.5 (2)	C2—C3—C4—C5	−0.5 (2)
O4—Na1—O5—Na1^ii^	−174.08 (4)	C2—C3—C4—C7	179.01 (14)
O3—Na1—O5—Na1^ii^	−85.38 (4)	C3—C4—C5—C6	0.5 (2)
O4^i^—Na1—O5—Na1^ii^	100.98 (4)	C7—C4—C5—C6	−179.03 (14)
O5^ii^—Na1—O5—Na1^ii^	0.0	C4—C5—C6—C1	−0.2 (2)
Na1^i^—Na1—O5—Na1^ii^	143.33 (4)	C2—C1—C6—C5	−0.2 (2)
O2—S1—C1—C6	−37.45 (12)	S1—C1—C6—C5	−175.66 (11)

Symmetry codes: (i) −*x*+1, −*y*+1, −*z*; (ii) −*x*+1, −*y*+2, −*z*.

### Hydrogen-bond geometry (Å, °)

**Table:**

Cg is the centroid of the C1–C6 ring.

**Table:**

*D*—H···*A*	*D*—H	H···*A*	*D*···*A*	*D*—H···*A*
O3—H31···O1	0.81 (1)	1.98 (1)	2.7885 (14)	173.(2)
O3—H32···O2^iii^	0.81 (1)	2.02 (1)	2.8059 (16)	166.(2)
O4—H41···O2	0.80 (1)	2.16 (1)	2.9038 (15)	154.(2)
O4—H42···O3^iv^	0.81 (1)	1.99 (1)	2.7884 (15)	173.(2)
O5—H51···O1^v^	0.80 (1)	1.99 (1)	2.7760 (15)	167 (2)
O5—H52···O2^i^	0.80 (1)	2.16 (1)	2.9319 (15)	163.(2)
O6—H61···O1^vi^	0.80 (1)	2.21 (2)	2.9528 (17)	154 (2)
O6—H62···Cg^vii^	0.79 (1)	2.89 (2)	3.3782 (17)	122 (2)

Symmetry codes: (iii) *x*, *y*+1, *z*; (iv) −*x*+1, *y*−1/2, −*z*+1/2; (v) −*x*+1, *y*+1/2, −*z*+1/2; (i) −*x*+1, −*y*+1, −*z*; (vi) *x*, −*y*+3/2, *z*−1/2; (vii) *x*, −*y*−1/2, *z*−3/2.
